# Assortment of carbon sources in medium for *Yarrowia lipolytica* lipase production: A statistical approach

**DOI:** 10.1007/s13213-014-0988-7

**Published:** 2014-10-11

**Authors:** Agata Urszula Fabiszewska, Danuta Kotyrba, Dorota Nowak

**Affiliations:** 1 Department of Chemistry, Faculty of Food Sciences, Warsaw University of Life Sciences, Nowoursynowska Street 159c, 02-787 Warsaw, Poland; 2 Department of Fermentation Technology, prof. Wacław Dąbrowski Institute of Agricultural and Food Biotechnology in Warsaw, Rakowiecka Street 36, 02-532 Warsaw, Poland; 3 Department of Food Engineering and Process Management, Faculty of Food Sciences, Warsaw University of Life Sciences, Nowoursynowska Street 159c, 02-787 Warsaw, Poland

**Keywords:** Glycerol, Mixture design, Lipolytic activity, Lipase, *Yarrowia lipolytica*

## Abstract

Glycerol is considered an important renewable feedstock as well as an undesirable side-product of biodiesel production. The aim of this study was to determine whether supplementing a culture medium with a combination of three different carbon sources (olive oil, glucose and glycerol) would optimize lipase production by the yeast *Yarrowia lipolytica*. The optimization experiments were conducted with a statistical approach using the mixture design. Analysis of the response surface revealed that it would be possible to compose a medium in which both an an extracellular lipase activity of 0.1 U/mL and up to 37.5 g/L of pure glycerol could be obtained. An YPO-Gl30 medium consisting of 30 g/L glycerol and 19.2 mL/L olive oil was selected for further investigation. Although a high biomass yield was found in all cultures, the glycerol content of the YPO-Gl30 medium slightly influenced yeast growth, but it did not prolong the duration of the lag phase. The hydrolytic activity of the extracellular lipases produced in YPO-Gl30 medium was satisfactory.

## Introduction

Microbiological research places great importance on the use of microorganisms for environmental protection. Such applications often involve the use of specific microorganisms to colonize contaminated environments and attempts to bioconvert waste residues into valuable products. The ability of various bacteria, yeast and fungi to grow on specific carbon and energy sources has led to their use in fermentation systems that utilize waste products. The application of “clean technology” in residue utilization and recycling is also dictated by the characteristics of culture media, including low cost, wide accessibility and a significant source of nutrients (Beopoulos et al. 2009; Salihu et al. 2012).

Glycerol is considered to be an important renewable feedstock as well as an undesirable side-product various industrial processes (Makri et al. 2010). The main industrial source of crude glycerol is the fuel industry, in particular biodiesel production (Rywińska et al. 2013). Attempts have been made to use microbial fermentation systems to convert glycerol into 1,3-propanediol, as well as to produce mannitol and erythritol and synthesize invertase. Among the frequently discussed microorganisms with a great potential for applications in future biotechnologies is the yeast species *Yarrowia lipolytica* (Rywińska et al. 2013)*.*

*Yarrowia lipolytica* is the only species within the genus *Yarrowia* and an important organism for many industries because of its high secretory activity, including citric acid, γ-lactones and enzymes such as protease, RNase, phosphatase, esterase and lipase. *Y. lipolytica* is commonly found in environments contaminated with hydrocarbons and oily wastes (Barth and Gaillardin 1997; Fickers et al. 2005; Kurtzman et al. 2011; Groenewald et al. 2013; Brigida et al. 2014). This yeast is also known for its ability to utilize lipid substrates, including waste oils (Bankar et al. 2009), as well as pure and crude glycerol (Rywińska et al. 2013) to produce a number of valuable metabolites. Rywińska et al. (2013) reported that this yeast can synthesize single cell protein (SCP), single cell oil and organic acids, including citric acid, in media containing glycerol. In 2009, the Polish company Skotan SA in collaboration with the Wroclaw University of Life Sciences started the industrial production of *Y. lipolytica* biomass feed (SCP) using glycerol waste from biodiesel production. Noteworthy, the company managed to register the feed in the European Food and Safety Authority (Rywińska et al. 2013).

A number of attempts have been made to optimize synthetic media and media with agroindustrial residues for microbial lipase production (Fickers et al. 2006; Treichel et al. 2010; Salihu et al. 2012). Lee et al. (2007) reported that *Y. lipolytica* NRRL Y-2178 is capable of alkaline lipase synthesis when glycerol is used as a carbon source. An interesting solution was presented by the Belgian spin-off company Artechno, which co-produced a freeze-dried yeast biomass and extracellular lipases of *Y. lipolytica* in a culture medium containing waste materials as the carbon source (Fickers et al. 2005).

In a recent study by Fabiszewska et al. (2014), wild-type strain *Y. lipolytica* KKP 379 was cultured in medium containing glycerol as the sole carbon source and the synthesis of lipolytic enzymes determined. The authors concluded that glycerol utilization in microbiological lipase production is indeed possible, but that this process can not proceed without the addition of one or more stimulators of lipase synthesis, such as olive oil.

 The aim of the study reported here was to apply an unconventional statistical approach to investigate the possibility that the addition of three different carbon sources to a culture medium would stimulate lipase production by *Y. lipolytica* KKP 379. The three carbon sources selected were olive oil, glucose and glycerol, and the optimal relative proportions of those substrates were evaluated in Mixture Design experiments. Glucose is a common carbon source for microorganisms, but extracellular lipase activity of *Y. lipolytica* has been found to be relatively low in the presence of glucose and only achieved after depletion of this substrate from the culture broth (Fickers et al. 2003). This led to our choice of also testing other carbon substrates. Olive oil is recognized as the best inducer for lipase production by yeast cells (Barth and Gaillardin 1997; Darvishi et al. 2009). Glycerol is considered to be an alternative to glucose when used in combination with an activator of lipase synthesis. We assumed that a specific concentration of glycerol in a culture medium for *Y. lipolytica* would not exhibit a repressive nature. The experiments presented here are preliminary and, therefore, pure glycerol was tested rather than crude glycerol.

## Materials and methods

### Chemicals

*p*-Nitrophenyl laurate was synthesized in our laboratory (Vogel et al. 1996). Glucose, peptone and yeast extract were purchased from BTL (Łódź, Poland) and pure glycerol (purity 99.5–100 %) was purchased from Chempur (Piekary Śląskie, Poland). We used commercially available extra virgin olive oil.

### Microorganism

*Yarrowia lipolytica* KKP 379 was purchased from the Collection of Industrial Microorganisms at the prof. Wacław Dąbrowski Institute of Agricultural and Food Biotechnology in Warsaw and was stored in liquid nitrogen. Yeast biomass was characterized by dry cell mass (d.m.) measured by the thermogravimetric method at 105 °C.

### Culture media and batch processes

Media in 100-mL shake culture flasks were supplemented with the chosen (combination of) carbon source(s) (glucose, glycerol and/or olive oil) in amounts according to three-factor mixture design for six factor levels (Table 1) because one of the aims of the experiment was to model the blending surface so that predictions of the response for any mixture component, alone or in combination, could be made. Two independent experimental schemes were designed based on different assumptions regarding the maximum amount of carbon sources selected: for glucose, 20.0 and 30.0 g/L; for olive oil, 24.0 and 36.0 mL/L; for glycerol, 150.0 and 225.0 g/L. Three additional medium were tested: YPG (20.0 g/L glucose), YPGO (20.0 g/L glucose and 12.0 mL/L olive oil) and YPO-Gl30 (19.2 mL/L olive oil and 30.0 g/L glycerol). All culture media, irregardless of the carbon source(s), contained 10.0 g/L yeast extract and 20.0 g/L peptone and had a pH of 5.0.

**Table 1:** The composition of carbon sources used in culture media for *Yarrowia lipolytica*

Medium	Pseudo components (%)^a^		
	Glucose	Olive oil	Glycerol
I experimental scheme (the composition of culture media designed for low levels of the maximum amount of carbon source)			
1	100	0	0
2	0	100	0
3	0	0	100
4	50	50	0
5	50	0	50
6	0	50	50
7	33.3	33.3	33.3
8	66.7	16.7	16.7
9	16.7	66.7	16.7
10	16.7	16.7	66.7
II experimental scheme (the composition of culture media designed for high levels of the maximum amount of carbon source)			
1 (11)	100	0	0
2 (12)	0	100	0
3 (13)	0	0	100
4 (14)	50	50	0
5 (15)	50	0	50
6 (16)	0	50	50
7 (17)	33.3	33.3	33.3
8 (18)	66.7	16.7	16.7
9 (19)	16.7	66.7	16.7
10 (20)	16.7	16.7	66.7
The composition of culture media designed for validation of a model calculated from experiments according to the I experimental scheme			
1w	0.5	0.4	0.1
2w	0.1	0.72	0.18
The composition of culture media designed for validation of model calculated from experiments according to the II experimental scheme			
3w	0.5	0.4	0.1
4w	0.05	0.9	0.05

^a^A pseudo component is the content of that component expressed as a percentage of the maximum content of that ingredient chosen for the experimental design: e.g. the maximum content of glucose in the I mixture design is 20.0 g/L, so its content in an amount of 10.0 g/L is expressed as a pseudo component of 50 %

The yeast cells were cultivated on a rotary shaker (200 rpm) in 500-mL Erlenmeyer flasks containing 100 mL of medium at 28 °C for 65 h. *Y. lipolytica* was also cultivated in a batch culture for 40–42 h in a 5-L bioreactor Bioflo 3000 (New Brunswick Scientific, Edison, NJ), a working volume of 4 L, at 28 °C, 300 rpm agitator speed and 0.0375 % (v/v) inoculum. Medium was aerated with compressed air at a flow of 105 L/h per 1 L medium. The biomass from the bioreactor was separated by centrifugation at 6,784 *g* for 10 min at 10 °C and washed with sterile 0.85 % NaCl solution. Precultures of *Y. lipolytica* were prepared in 500-mL Erlenmeyer flasks, each containing 100 mL YPG medium, and incubated for 24 h at 28 °C and 150 rpm rotary shaker speed. Inoculum for the flasks and bioreactor was standardized by measuring the optical density of the culture.

### Determination of lipolytic activity

Measures of enzymatic activity were carried out using a modified spectrophotometric method based on the hydrolysis of *p*-nitrophenyl laurate (molar extinction coefficient 0.01795 cm^2^/μM) (Krzyczkowska et al. 2009; Kapturowska et al. 2012). One unit of lipase activity was defined as the enzyme quantity that liberated 1 μmol of *p*-nitrophenol per minute under the assay conditions at 37 °C and pH 7.0.

### Medium optimization by the statistical approach

Due to the need to perform multiple combinations of three selected substrates, we used the mixture design method of DoE (design of experiment) as a tool to facilitate optimizing the composition of carbon sources in the culture medium (Mason et al. 1989). The statistical package Statistica 10.0 (StatSoft, Tulsa, OK) was used to generate mixture experiments. The significance level was 0.05. Two separate experimental schemes and two parallel cultures for each design were prepared based on simplex designs for ternary mixtures. The schemes varied in terms of the assumptions regarding the maximum amount of carbon source(s). Within a particular experimental scheme it was possible to evaluate three different mixture designs: simplex lattice design (medium 1–6, Table 1), simplex-centroid design (medium 1–7, Table 1) and simplex extended design (medium 1–10, Table 1).

The procedure of analysis consisted of evaluating several models of the blending surface and formulating a suitable one. The models were fitted for two independent variables: biomass yield and extracellular activity of *Y. lipolytica* lipases. An analysis of variance was performed in order to check the significance and fitness of the models. The reliability of the models was evaluated by calculating the determination coefficients [square of the respective correlation coefficient (*R* ^2^)] and the validation coefficients *Q* ^2^ values for each model, where *R* ^2^ is the variation of the response explained by the model and *Q* ^2^ is the fraction of the response variation that can be predicted by the model. Validation was performed by the cross-validation method described by Mazerski (2009). In addition, the adequacy each model was evaluated by comparing the data approximated from that model with values from experiments which were carried out using additional design points (media 1w, 2w, 3w and 4w, Table 1).

## Results

### Fitting an appropriate mathematical model in a mixture experiment

Optimization of the composition of the carbon source in the culture medium for stimulating the synthesis of yeast lipases was performed on a laboratory scale in 100-mL shake culture flasks. Variability of extracellular lipolytic activity and biomass yield were described using analytic functions. The contents of three different carbon sources were the components of the mixture, and the remaining ingredients were used in constant amounts.

The choice of maximum levels for carbon sources, mainly glucose and olive oil, were based on the amounts previously used in our laboratory. The glycerol concentration in the medium was based on the results of preliminary studies (Fabiszewska et al. 2014) carried out in our laboratory and was not in accordance with that reported by other authors. The results of our own studies confirmed the need to use higher concentrations of glycerol relative to the other two sources of carbon. A high content of glycerol as a sole carbon source was also used by Makri et al. (2010) in the medium for *Y. lipolytica* ACA-DC 50109 (105 g/L). In turn, Wang et al. (2008) studied the effect of olive oil content on the lipase activity of *Rhizopus chinensis* and reported that 20 g/L was the optimum olive oil concentration in terms of maximizing the lipolytic activity of this mold. Increasing the content of olive oil to either 30 or 40 g/L resulted in decreased lipase activity associated with the cell wall of up to approximately 60 and 35 %, respectively (Wang et al. 2008). The results of our preliminary study were in accordance with those of Wang et al. (2008), and we therefore tested olive oil at maximum concentrations of 20 and 30 g/L (24.0 and 36.0 ml/L, respectively).

*R* ^2^ relates to the variance of the model and is considered to be a better measure than the correlation coefficient. The validation coefficient ( *Q* ^2^) refers to the variation of the response and can be calculated from the model. A good model should be characterized by high values of both coefficients (>0.5), and a perfect model has *R* ^2^ and *Q* ^2^ values of close to unity. Moreover, a good model should have the lowest possible difference between the values of both of these factors (Mazerski 2009).

*R* ^2^ and * Q* ^2^ for models calculated based on the three different experimental designs are shown in Table 2. The models were formulated on the basis of non-significance of the lack of fit. If the lack of fit was non-significant, the proper model was selected on the basis of additional criteria: a high value of * R* ^2^ and * Q* ^2^ statistics. The lack of fit was significant only for two models (selected on the basis of experiments using simplex extended designs) and these models were therefore not included in further analyses (Table 2).

**Table 2:** Determination and cross-validation coefficients for the calculated models for the two variables biomass yield and extracellular lipase activity^a^

Determination and cross-validation coefficients	Design of experiment					
	Simplex lattice		Simplex centroid		Simplex extended	
	Biomass yield	Lipase activity	Biomass yield	Lipase activity	Biomass yield	Lipase activity
I experimental scheme						
Determination coefficient (*R* ^2^)	0.8546	0.9143	0.7012	0.8883	–	0.8267
Cross-validation coefficient (*Q* ^ 2^)	0.4832	0.9212	0.4227	0.8546		0.7012
II experimental scheme						
Determination coefficient (*R* ^2^)	0.7308	0.9002	0.7352	0.8923	0.4730	–
Cross-validation coefficient (*Q* ^ 2^)	0.1910	0.7761	0.3190	0.7638	0.5825	

^a^Models were calculated from experiments based on three simplex designs

Analysis of *R* ^2^ and *Q* ^2^ allowed the selection of an appropriate mathematical model for extracellular lipase activity that was best suited to the experimental data and had good predictive abilities. The model based on the simplex lattice design in the I experimental scheme was fitted by >91 % to the observed data (*R* ^2^ = 0.9143; Table 2), meaning that >90 % of the variation in lipase activity was attributable to the independent variables. The selected model was also characterized by its good prognostic abilities, which was confirmed by the cross-validation coefficient (*Q* ^2^ = 0.9212). Some components of this function were removed because they did not have a statistically significant impact on the variability and therefore repeated some of the information provided by the remaining variables in the model. Therefore, the following model was developed for extracellular lipolytic activity:

$$ A=0.000925\times x+y+0.0077083\times 0.000034\times z-0.0004775\times x\times y-8.56111\times {10}^{-5}\times y\times z $$

where *A* is extracellular lipase activity (U/mL), *x* is the amount of glucose in a medium (g), *y* is the amount of olive oil in a medium (mL), and *z* is the amount of glycerol in a medium (g). The canonical polynomial of degree one was formulated for a three-component mixture, where mixture proportions for the three components, denoted *x*,* y*,* z*, were such that* x* + *y* +* z* = 1.

In the case of biomass yield, two models proved to accurately fit with the experimental data: a model calculated from the simplex lattice design in the I experimental scheme (*R* ^2^ = 0.8546; Table 2) and a model calculated from the simplex centroid design in the II experimental scheme (*R* ^2^ = 0.7352). However, due to low predictive quality of both models, a model was chosen based on a simplex extended plan in the II experimental scheme (Table 2), for which a distinction between coefficients of determination and validation were the lowest (*R* ^2^ = 0.4730 and *Q* ^2^ = 0.5825). A mathematical model took the form of a linear function:

$$ P=0.35\times 0.544\times x+y+0.04133\times z $$

where *P* is biomass yield (g d.m./L), *x* is the amount of glucose in the medium (g), *y* is the amount of olive oil in a medium (mL) and *z* is the amount of glycerol in the medium (g).

The model selected for biomass yield was characterized by weaker prognostic capabilities relative to the model identified for lipolytic activity. It would appear that factors other than the type of carbon source were able to significantly determine the final biomass yield. We did not take these factors, such as physiological state of the yeast cells in inoculation preculture, into account in the medium optimization experiments.

In order to validate the prediction models, we carried out additional shake culture experiments in 100 mL of medium 1w, 2w, 3w and 4w (Table 1) inoculated with *Y. lipolytica*. Based on the results (Table 3), the lowest average difference in lipase activity was obtained using the model based on a simplex lattice design (I experimental scheme). In the case of biomass yield the model based on the extended simplex design (II experimental system) was confirmed to be the best fitted model. Experimental validation for biomass yield did not support the cross-validation results. For the I experimental scheme, the smallest average differences between the experimental data and the values approximated from the models were achieved for the simplex lattice and simplex centroid designs (2.50 and 1.80 g d.m./L, respectively; Table 3). In contrast, among the three models for the II experimental scheme, the smallest difference (7.33 g d.m./L) characterized a model calculated on the values from the simplex extended design.

**Table 3:** Average differences between the experimental data and the data approximated from a particular model for biomass yield and extracellular lipase activity

Lipase activity/biomass yield	Design of experiment		
	Simplex lattice	Simplex centroid	Simplex extended
Extracellular lipase activity (U/mL)			
I experimental scheme	0.0117 ± 0.0226	0.0193 ± 0.0212	0.0119 ± 0.0196
II experimental scheme	0.0199 ± 0.0128	0.0781 ± 0.0330	–
Biomass yield (g d.m./L)			
I experimental scheme	2.50 ± 0.84	1.80 ± 1.06	–
II experimental scheme	9.60 ± 1.44	9.36 ± 1.50	7.33 ± 2.75

The results are presented as the average ± standard deviation (SD)

### Selection of the best culture medium composition for lipase production

**Fig. 1:**
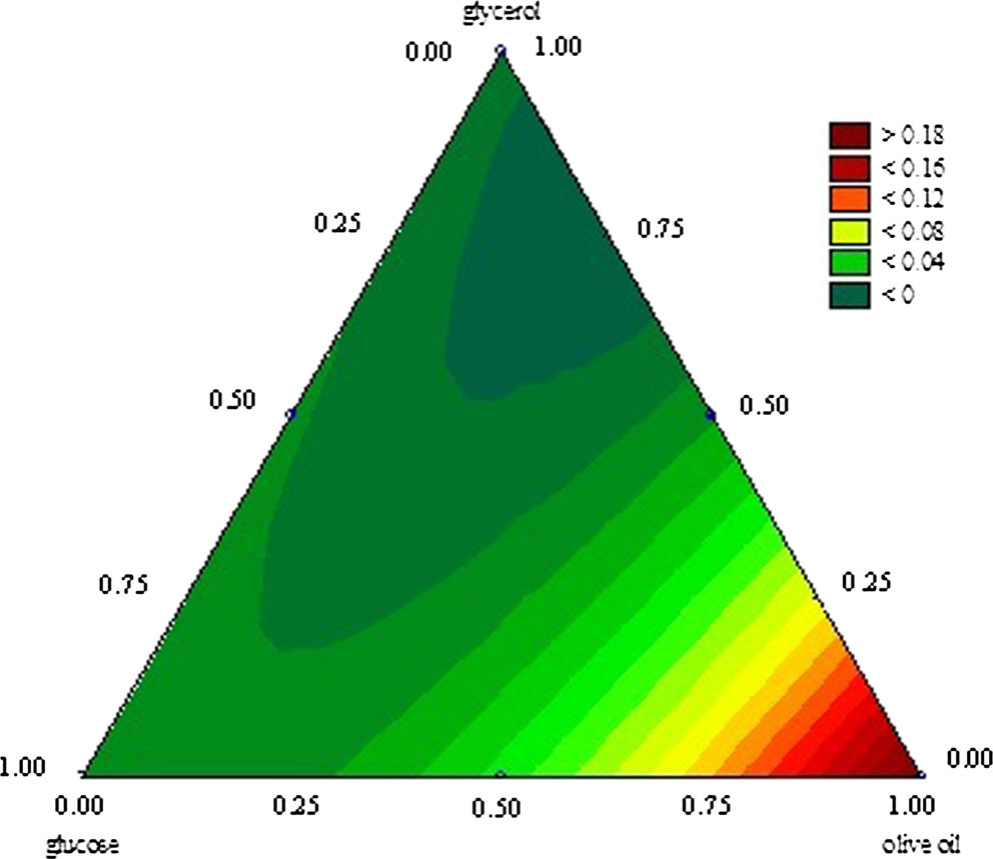
Contour plots of the blending surface for extracellular lipase activity of *Yarrowia lipolytica* (U/mL) according to the composition of carbon sources

**Fig. 2:**
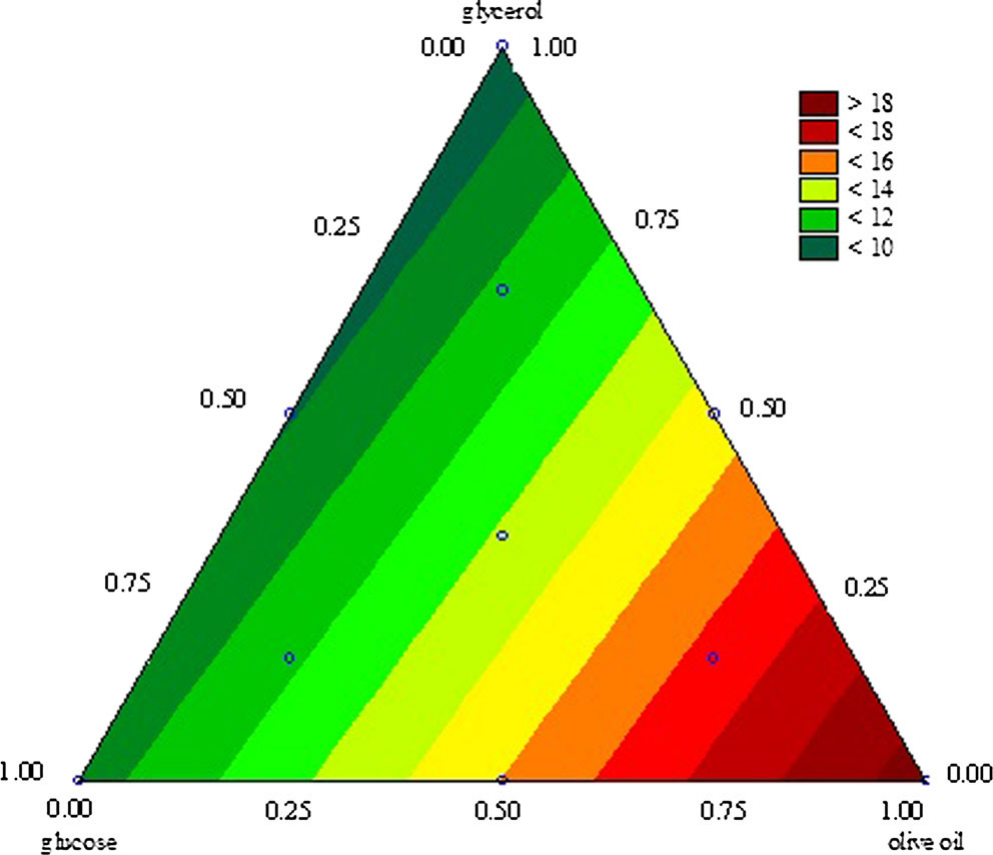
Contour plots of blending surface for yeast *Y. lipolytica* biomass yield (g d.m./L) according to the composition of carbon sources

The relation between the process variables can be observed in the plots of Figs. 1 and 2. The experimental region of interest in the three-component mixture experiment was defined by values of mixture proportions in a regular equilateral triangle. Vertices of the triangle represent the individual components, and points on the each side of the triangle represent the binary blends. We used the plots to determine the composition of the three carbon sources in the medium which would allow both a satisfactory biomass yield (Fig. 2) and a high extracellular lipase activity of *Y. lipolytica* (Fig. 1) to be achieved in a medium with a high proportion of glycerol. The results presented on Fig. 1 confirm that olive oil acted as an inducer of lipase activity and that the presence of glycerol and glucose had a negative effect on the extracellular lipase activity of *Y. lipolytica*. We determined that the best medium for extracellular lipase production by the yeast was a monoculture with olive oil. Similarly, the biomass yield was higher in media containing from 50–100 % of the assumed maximum content of olive oil than in media containing either or both of the other substrates (Fig. 1). Less satisfying results for biomass yield were achieved in the medium containing glycerol as 100 % of the carbon source, and the highest yeast biomass was achieved in monoculture with olive oil (Fig. 2). At the same time the analysis of surface response showed that it was possible to compose a medium that would concomitantly facilitate an extracellular lipolytic activity of 0.1 U/mL and contain up to 25 % of the assumed maximum content of glycerol (37.5 g/L).

Biomass yield and enzyme activity are usually higher under high oxygenation conditions (e.g. aerated bioreactor culture) than in shake flask culture. Cultivating the culture in a bioreactor was therefore important due to future potential applications. A YPO-Gl30 medium[30 g glycerol (20 %) and 19.2 mL olive oil (80 %)] was used for the bioreactor experiments. However, it was a challenge to transfer the results to a larger scale due to differences in heat transport and mass transfer, as well as differences in the method of shaking which might have caused shear stress, thereby disrupting cell growth.

### Lipase production in a laboratory-scale bioreactor

The results found using the YPO-Gl30 medium were compared with those using the YPGO medium commonly used in *Y. lipolytica* culture in our laboratory (Table 4). We found that comparable extracellular lipase activity was achieved in both media [0.141 (YPGO) vs. 0.135 U/mL (YPO-Gl30); Table 4]. The activity of cell-bound lipases in the bioreactor culture was also satisfactory for these two media [10.17 (YPGO) vs. 9.19 U/g d.m. (YPO-GI30)]. Although a high biomass yield was observed in all cultures [24.7 (YPGO) vs. 18.9 g d.m./L (YPO-Gl30)], the glycerol present in the YPO-Gl30 medium slightly influenced yeast growth, but did not prolong the duration of the lag phase. It may thus be concluded that the catalytic properties of *Y. lipolytica* in the medium optimized in the mixture design experiment were similar to those achieved in the YPGO medium.

**Table 4:** Biomass yield and lipase activity of *Y. lipolytica* in YPGO and YPO-Gl30 medium-based bioreactor culture

Medium^a^	Carbon source composition	Duration of lag phase (h)	Biomass yield (g d.m./L)	Extracellular lipase activity (U/mL)	Cell-bound lipase activity (U/g d.m.)
YPGO	20 g/L glucose 12.0 mL/L olive oil	14.25 ± 0.25	24.7 ± 1.4	0.140 ± 0.02	10.17 ± 1.93
YPO-Gl30	30 g/L glycerol 19.2 mL/L olive oil	11.25 ± 0.25	18.9 ± 0.4	0.135 ± 0.02	9.19 ± 1.11

d.m., Dry cell mass

The results are presented as the average ± standard deviation (SD)

All culture media, irregardless of the carbon source(s), contained 10.0 g/L yeast extract and 20.0 g/L peptone and had a pH of 5.0. YPGO medium contained 20.0 g/L glucose and 12.0 mL/L olive oil; YPO-Gl30 medium contained 19.2 mL/L olive oil and 30.0 g/L glycerol

## Discussion

The production of lipolytic enzymes in media with glycerol has not been studied to a great extent mainly because *Y. lipolytica* cells can use glycerol as a carbon source to produce high amounts of organic acids, such as citric, isocitric and 2-ketoglutaric acids (Makri et al. 2010; Rywińska and Rymowicz 2010) and erythritol (an important natural sweetener; Mirończuk et al. 2014). Darvishi et al. (2009) suggested that lipase and citric acid could be produced simultaneously by *Y. lipolytica* DSM 3286 using plant oils. In all of the media tested, these authors observed maximum lipase activity after 48 h of growth and a high level of organic acid production after 72 h. Fabiszewska et al. (2014) recently reported that *Y. lipolytica* KKP 379 did not show such abilities in media containing pure glycerol as the carbon source . Glycerol is a hydrolysis product of triglyceride and can inhibit the production of lipases, as has been suggested for *Candida rugosa* lipase production [same class (*Saccharomycetes*) and order (*Saccharomycetales*) as *Y. lipolytica*]. Dalmau et al. (2000) reported that *C. rugosa* lipases synthesized on glycerol medium were inactive. Del Rio et al. (1990) and Kamzolova et al. (2011) also investigated the inhibitory action of glycerol on the lipase activity of *C. rugosa*. These authors reported that the activity of *Y. lipolytica* extracellular lipase began to increase just from the start of yeast growth and remained at a high level (approximately 10 U/mg cells) during the whole cultivation period on rapeseed oil medium. However, when the yeast was cultivated on glycerol, the activity of lipase began to rise only when the concentration of glycerol fell to 4.0 g/L. In this case, the activity of lipase did not exceed 3.0–3.2 U/mg cells (Kamzolova et al. 2011).

It has been confirmed that *Y. lipolytica* cells can selectively use fatty acids (Papanikolaou et al. 2003), and data reported in the literature suggest that free fatty acids present in oils are good inducers of lipase synthesis due to their high content of oleic acid. The addition of vegetable oils or free fatty acids to a culture medium is a common practice in microbiological lipase synthesis (Fickers et al. 2005, 2011). Kamzolova et al. (2005) reported that olive oil is the best activator of lipase activity in *Y. lipolytica* 704, as confirmed in our own results for *Y. lipolytica* KKP 379. Galvagno et al. (2011) presented an optimization of biomass production of a genetically modified strain of *Y. lipolytica*, NRRL Y-1095, grown in a medium with glycerol. These authors applied a Plackett–Burman design, a central composite design (CCD) and a response surface methodology and concluded that the addition of an organic nitrogen source and 1 % of olive oil was necessary to stimulate the synthesis of lipases. In their system, the maximum biomass yield and maximum activity of extracellular lipases were 17.1 g/L and 12.2 U/mL, respectively when the medium contained 13 g/L glycerol and 10 g/L peptone. In turn, Volpato et al. (2008) observed the maximum lipolytic activity of *Staphylococcus caseolyticus* when the bacterial cells were cultivated in a medium containing 30 g/L glycerol and 3 g/L olive oil.

Makri et al. (2010) reported that the synthesis of *Y. lipolytica* lipases began at the time-point when >95 % of the initial glycerol in the medium had been utilized. Galvagno et al. (2011) also observed an increase in the lipolytic activity of *Y. lipolytica* in the stationary phase of growth (36 h)—when the glycerol used as a carbon source had been exhausted. Complementary to these results may be observations of Corzo and Revah (1999), who found that the addition of 0.5–6.0 g/L glycerol to the medium did not significantly inhibit the synthesis of extracellular lipase by *Y. lipolytica* 681. However, the authors did confirm the thesis that a certain amount of glycerol in the culture medium may not have a repressive nature in relation to the production of lipolytic enzymes. These conclusions are in accordance with our results, i.e. it is possible to compose a medium containing glycerol (not more than 37.5 g/L) to cultivate *Y. lipolytica* with extracellular lipase activity at the level of 0.1 U/ml. It should be noted that the addition of lipase inducer, such as olive oil, is indispensable.

Papanikolaou et al. (2003) and Kamzolova et al. (2008, 2011) observed that when both glycerol and fatty acids are available in the medium they do not suppress the metabolism of each other. Moreover, the utilization of these two substrates was found to occur concurrently, although glycerol was utilized at a higher rate than oleic acid. In contrast, upon the cultivation of *Y. lipolytica* on the mixture of glucose and oleic acid, the latter substrate began to be utilized only when the concentration of glucose decreased significantly (Kamzolova et al. 2011). A similar inhibitory effect of glucose was observed for *Y. lipolytica* KKP 379 when the carbon source decreased lipolytic activity in comparison with olive oil. It should be also mentioned that so far there is no universal method for determining the activity of triacylglycerol hydrolases. Consequently, we were unable to compare our results taking the values of lipolytic activity into account, but we were able to compare the final effect observed in each study.

Interestingly, Thompson and He (2004) performed a detailed analysis of the composition of crude glycerol from biodiesel production when various vegetable oils (such as canola, rapeseed and soybean oils) as well as frying oils were used as carbon sources. The crude glycerol contained between 75 and 83 % pure substance on average and between 1 and 13 % of fats. A future experiment in which raw glycerol is used in the culture of *Y. lipolytica* KKP 379 seems promising because the fatty acids, which are a residue in the crude glycerol, might act as inducers of lipase expression. Such a possibility supports the results of Galvagno et al. (2011), who used a partially purified glycerol at an amount of 2.4 % (v/v) and achieved a comparable lipase activity of *Y. lipolytica* as in a medium containing 0.1 % (v/v) of olive oil.

The use of waste residues from the agricultural industry as a feedstock for the synthesis of lipases has a great advantage, namely, cost reduction. However, it should be carefully investigated whether such an approach reduces the global costs of the process, including the costs associated with the increased scale of microorganism culture and—in some cases—the cost of the additional enzyme purification steps. Sometimes, the use of waste products makes the process of purifying the lipase much more laborious. Furthermore, media containing waste feedstock often require additional supplementation with some nutrients or metabolic activity stimulators (Treichel et al. 2010). These aspects should be taken into account when deciding upon whether to transfer the technology of microbial enzyme production using waste materials from science to practice.

The use of a statistical approach in our study deserves comment. One of the main challenges when attempting to optimize culture conditions for microorganisms is to determine the effect of the many factors that can influence the growth of microbial cells or the synthesis of the desired metabolites, as well as the persistence of many complicated interactions between these agents. The traditional approach is to search for the optimal value of the parameter, while all other relevant conditions remain constant. These practices are very laborious and cost-intensive and do not allow examination of the mutual interactions between factors affecting the tested feature. This approach was used by, for example, Gulati et al. (1999), who separately optimized eight different factors affecting the synthesis of lipase in the culture of *Aspergillus terreus*. The need to perform several repetitions for each variant meant that the authors expended a tremendous effort for each experimental result. Unfortunately, they did not examine whether the use of all optimized culture parameters, which had been obtained separately, actually resulted in the highest possible lipolytic activity of *A. terreus* (Gulati et al. 1999). As a result, incorrect conclusions might have been drawn. Therefore, sophisticated statistical methods, such as DoE, are used in the field of biotechnology, as these allow the simultaneous optimization of many parameters and consequently lead to a significant reduction in the number of experiments (Panesar 2008; Teng and Xu 2008; Wang et al. 2008).

The statistical methods of DoE provide a systematic and efficient plan for experimentation, so that many factors influencing a process can be simultaneously studied. In recent years, statistical approaches have become useful tools for understanding the interactions among various parameters with a minimum number of experiments (Gupta et al. 2007). The DoE methods have many benefits compared to the conventional one-factor-at-a-time methods, which can fail to locate optimum parameters because it is impossible to describe a possible effect of interactions between the factors (Gupta et al. 2004; Teng and Xu 2008).

Previous attempts to apply statistical methods, including various regression methods and the design of the experiment, have made it possible to minimize the costs and maximize the effects in studies aimed at optimizing the esterification reaction catalyzed by lipases (Manohar and Divakar 2004; Adnani et al. 2010) and the biomass production of whole-cell biocatalyst *R. chinensis* characterized by high lipolytic activity (Teng and Xu 2008). DoE methods have also been applied to compose a medium for a new lipolytic strain of *Burkholderia multivorans* (Gupta et al. 2007) or to optimize the synthesis of extracellular lipase from *Geotrichum* sp. (Burkert et al. 2004) and *Candida* sp. (He and Tan 2006). In most cases, the authors of the studies used a factorial Plackett–Burman design and CCD (Treichel et al. 2010). To the best of our knowledge, our application of a mixture design is the first of its kind in terms of optimizing microbial medium composition. We conclude that it is a suitable method to determine which mixture or monoculture is most effective, productive or economically advantageous.
